# Address of Welcome of Major John J. McCann to the Members and Delegates at Nashville on Behalf of the State and the Tennessee Centennial

**Published:** 1897-11

**Authors:** 


### ADDRESS OF WELCOME OF MAJOR JOHN J. McCANN TO THE MEMBERS AND DELEGATES AT NASHVILLE ON BEHALF OF THE STATE AND THE TENNESSEE CENTENNIAL.

It is always a pleasure to me to extend the hospitality of my fellow-citizens and the management of the Tennessee Centennial and the citizens of the United States, and I assure you that I feel an extraordinary pleasure in welcoming you within the confines of our State.

There is no animal which confers so many blessings upon mankind as the horse, and I feel happy because I am permitted to address a class of gentlemen whose aim and object in life is not only to relieve his pains, but to release all the penalties to which horse-flesh is subjected.

I have been over fifty years in preparing the impromptu speech which I make you to-day.

If there is any subject on earth with which I am more familiar than any other, it is the horse. My earliest recollection is closely associated with the "clothes-horse," upon which I used to hang my clothes when I was a child, and one which saved my fingers from many a burning. With what boyish delight did I dwell upon my "rocking-horse." He was a "goer"—you bet he was. Like Alexander the Great, I found that my Bucephalus was afraid of his shadow; so I turned his head to face the sun, and he couldn't see it. Rarey, the great horse-tamer, wasn't in it with me. I sobered him down, so that in less time than it takes lightning to chase a squirrel down a hickory tree he was not even afraid of the tom-cat. Philip, the father, immediately said: "Seek, my son, another kingdom; Macedonia is unworthy to contain thee." Acting upon parental instructions, I struck a bee-line for a neighboring stream, where, with dangling line and a wriggling red-worm, I threw tempting bait to the red horse, "roped him in," and soon I found him dangling on my string.

The next thing was the "wood-horse." Gee whiz! How I sweated and toiled over this fellow! I had a strong constitution, stood a good deal of rest, and wound up with my head full of "wise-saws and modern instances."

The next thing I struck was the "sea-horse." He was more often heard of than seen in public. I never liked him very much, because I am built not to like any fellow who is fond of nothing stronger than water. (Laughter.)

Then there is the "horse-fly." I never had much passion for him. He is not easily shaken off. He is a "sticker," from pastern to withers; to be more comprehensive, I will say from snout to crupper-bone, and sometimes creates a disposition on the part of the horse to walk out from under the saddle and give us a new evidence of the "fall of man." (Applause.)

Then there is the "old horse," with his mane flowing to the breeze, his tail over the dashboard, splitting the road wide open, and teaching a gazing world that age ain't nothing; "it is blood which tells." How often have I seen the aged veterinarian strike a bee-line for a grocery, annihilating distance! Robert J., Hal Pointer, Iroquois, and other quick-steppers were all left in the distance as they passed the grand stand, and amid the shouts of the multitude you saw the veterinarian smile as he took down the purse which contained the price of his fee and received the congratulations of the horse-owner as he said: "The veterinarian is a sure winner."

The next fellow is the horse-trader, as he came charging down on me for a new deal. I cannot tell you his exact age, as he had worn his teeth off in champing the bit, so eager was he for the fray. But the slickest one in this land I ever knew was the boy-trader. He had a mule which he had trained so that when he touched him on the flank he would immediately sit down. He struck an Englishman one day from across the briny deep, who had come to this country to take him a glorious hunt. As he rode along he met a country boy, and as they sauntered along the lane together the boy espied a rabbit in the field. He immediately touched his mule in the flank, and the mule sat down. The Englishman asked what was the matter with the mule. The boy replied that he was a "setter" and he was setting the rabbit, and showed the Englishman the rabbit as he scampered away. A short distance further on the boy espied a partridge. He immediately touched the mule on the flank, and down he sat. The Englishman was overjoyed, bantered the boy for a horse-trade, and the boy finally got rid of his mule for the Englishman's horse and a hundred dollars to boot. They changed mountings, until a short distance further along they crossed a stream. The Englishman put his spurs into the flanks of the mule and urged him forward, when the mule immediately sat down. Says the Englishman: "What is the matter with him now? "Oh," says the boy, "he sets just as well for suckers as he does for rabbits and birds." (Loud applause.) In a word, that mule "went all the gaits." And the only reason my fellow-citizens seem to have in trotting me out to make addresses of welcome grows out of the fact that I talk to the boys, smile on the girls, and take another kind of smile with the boys. In fact, my tank-capacity has been equal to any show which comes our way.

Aside, now, gentlemen, from levity, I again welcome you to our city, and desire to say to you that you are monarchs of all you survey, and there is none your right to dispute. If there is anything you want which you do not see, let me know, and you shall have it. I am a brother-in-law in the Methodist Church—Dr. Haggard's church—and therefore can do no wrong. God bless every one of you. It is just such gatherings as we have to-day which add to the glow of that spirit of nationality so essential to the nation's prosperity. And while you attend to the business you have in hand, I will take the girls on a trolley ride and return them all to you in perfect safety. I was born in this Southland, where the first lessons we were taught by our mothers was to take care of the girls; let them have a good time and show them the elephant, tusks and all. And I know that I shall be amply repaid for my trouble by the sweet music which will flow out from this row of chimes you have brought with you. (Applause.) We will now move out, girls, and when we get back you will pronounce a benediction on the trip and say that you have been out with the handsomest "charger on the stretch." (Laughter.)

And the members of the press are here—the faithful chroniclers and motive-power of all associations, and with whom I have a contract to write all my speeches, will faithfully chronicle all that is done and said in my absence, and I will be sure to keep up with the procession. May Heaven bless you, newspaper boys! They have condoned my faults and magnified my virtues to such an extent that, while my dear, sweet wife don't regard me as a Solomon, she thinks I am no sardine, by a large majority. (Laughter.)

We have the lights from the great universities among us to-day, whose permeating beams will brighten our way through the midnight of gloom and doubt. Guided by our past experiences, and aided by such lights as these, how can we fail to succeed in relieving the pain which exists in the forest of the lumbar region of the noble horse. (Applause.)

